# Significant Tic Reduction in An Otherwise Treatment-Resistant Patient with Gilles de la Tourette Syndrome Following Treatment with Nabiximols

**DOI:** 10.3390/brainsci7050047

**Published:** 2017-04-26

**Authors:** Ahmad Seif Kanaan, Ewgeni Jakubovski, Kirsten Müller-Vahl

**Affiliations:** 1 Clinic of Psychiatry, Socialpsychiatry and Psychotherapy, Hannover Medical School, Carl-Neuberg-Str. 1, Hannover 30625, Germany; amadeus.kanaan@gmail.com (A.S.K.); jakubovski.ewgeni@mh-hannover.de (E.J.); 2 Max Planck Institute for Human Cognitive and Brain Sciences, Stephanstraße 1a, Leipzig 04103, Germany

**Keywords:** Tourette syndrome, tic disorders, nabiximols, endocannabinoid system

## Abstract

Early anecdotal reports and preliminary studies suggested that cannabinoid-based medicines such as delta-9-tetrahydrocannabinol (THC) are effective in the treatment of Gilles de la Tourette syndrome (TS). We report a single case study of a patient with otherwise treatment-resistant TS successfully treated with nabiximols. Our patient was a 22-year-old male suffering from severe and complex TS. Treatment with nabiximols was commenced at a dose of 1 puff/day (= 100 μL containing 2.7 mg THC and 2.5 mg cannabidiol (CBD)) and slowly increased up to a dosage of 3 × 3 puffs/day (= 24.3 mg THC and 22.5 mg CBD). Several clinical measures for tics, premonitory urges, and global impairment were acquired before and after two weeks of treatment. Treatment with nabiximols resulted in major improvements of both tics and premonitory urges, but also global impairment and health-related quality of life according to all used measurements without causing relevant adverse effects. Our results provide further evidence that treatment with nabiximols may be effective in the treatment of patients with TS. Given the positive response exhibited by the patient highlighted in this report, further investigation of the effects of nabiximols is proposed on a larger group of patients in a clinical trial setting.

## 1. Introduction

Gilles de la Tourette syndrome (TS) is a disorder defined by the presence of multiple motor tics and at least one vocal tic. TS can be found in about 1% of the general population [1,2], and is three to four times more prevalent in males than in females [3,4,5]. Despite the fact that the range of treatments available for tic disorders and TS is gradually expanding, treatments are often ineffective or cause significant side effects [6]. Therefore, there is an unmet need for more effective treatment options which cause fewer side effects and could potentially benefit otherwise treatment-resistant patients. Early anecdotal reports of successful self-medication with cannabis [7,8] provided evidence for an alternative mechanism of drug action that involves the central endocannabinoid system. Accordingly, cannabinoid-based medicine (CBM) such as delta-9-tetrahydrocannabinol (THC) has been suggested as such an effective new treatment strategy for patients with TS [9,10]. Nabiximols is a plant extract from *Cannabis sativa* L. containing THC and cannabidiol (CBD) at a 1:1 ratio. Anecdotal reports as well as preclinical data have provided some evidence that nabiximols may be more effective and better tolerated than pure THC, since CBD possesses its own effects [11] and mitigates the unwanted psychotropic effects of THC [12]. Nabiximols is formulated as an oromucosal spray and is partly absorbed sublingually, resulting in reduced first pass effects, faster absorption, accelerated onset, and stronger effect compared to orally administered pharmaceutical products such as pure THC [13]. To date there is only one published case study of a 26-year old male patient suffering from severe, otherwise treatment-resistant TS who improved significantly on nabiximols treatment [14]. In the light of this new development, further evidence is needed to judge the efficacy of this treatment route. To shed more light onto cannabinoid treatment of TS, we report a further case study of a 22-year old German patient treated with nabiximols.

## 2. A Case of Severe TS

Our patient is a 22 years-old male who lives in Germany. The patient has a family history of attention deficit/hyperactivity disorder (ADHD) and depression on the mother’s side, as well as a family history of tics from both parents. He reached all these developmental milestones in a timely fashion and suffered from meningitis and otitis media at the age of six years. He started exhibiting symptoms of ADHD and tics as early as elementary school. Despite his increasingly disturbing tics, he managed to finish nine years of high school and start an apprenticeship in a nursing program. However, due to further worsening of his tic severity, he was incapacitated and unable to finish his degree or to start another internship or training program. He presented in our clinic for the first time at age 21 with severe complex vocal tics such as pronounced coprolalia, echolalia and spitting, as well as severe complex motor tics such as copropraxia including touching himself or others, and self-injurious behaviors such as punching himself in the head as well as punching his father. Apart from the tics, the patient suffered from a number of comorbid symptoms and conditions such as obsessive-compulsive behaviors (need for symmetry, just-right-feeling), ADHD, sleep problems, flight anxiety and pathological gambling. The pathological gambling had eventually led to petty crime, upon which the patient had been convicted to a short prison sentence. During his time in prison, he had been physically attacked by his inmates because of his tics and needed to be transferred to a single cell. The severity of his TS pushed the patient to seek various available pharmacological treatments (e.g., haloperidol, risperidone, aripirazole, clonidine, methylphenidate, atomoxetine, fluphenazine, pramipexole) alone or in combination with different drugs at one time. All treatments were either ineffective or caused intolerable side effects. The patient had no relevant history of drug use.

## 3. Treatment with Nabiximols

We initiated treatment with nabiximols (Sativex®, GW Pharma limited, Cambridge, CB24 9BZ, UK) at a dose of 1 puff per day (= 100 µL containing 2.7 mg THC and 2.5 mg CBD) and slowly increased up to a dosage of 3 × 3 puffs per day (= 24.3 mg THC and 22.5 mg CBD). Clinical assessments were carried out twice, once before treatment with nabiximols and once after two weeks of a stable dose of 3 × 3 puffs per day. The following instruments used included: (1) the total tic score (TTS) of the Yale Global Tic Severity Scale (YGTSS) [15], Tourette Syndrome Symptom List (TSSL) [16], and Modified Rush Video-Based Tic Scale (MRVS) [17] for tics; (2) the Premonitory Urge for Tics Scale (PUTS) [18] and Global Clinical Impairment (GCI) for premonitory urges; (3) GCI and the global score (GS) of the YGTSS for global impairment; and (4) the GTS-Quality of Life (GTS-QOL) [19] and the Visual Analogue Scale for satisfaction of the GTS-QOL (GTS-QoL-VAS) for health-related quality of life.

**Figure 1:**
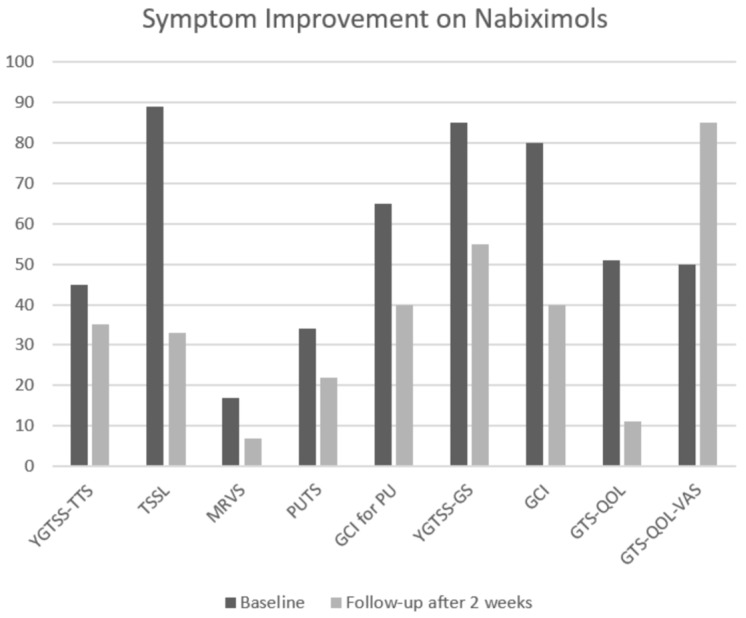
Symptom improvement with nabiximols. For GTS-QOL-VAS a lower score represents better quality of life; for all other scales a higher score is better. Abbreviations: YGTSS-TTS = total tic score of the Yale Global Tic Severity Scale (range: 0–50), TSSL = Tourette Syndrome Symptom List (range: 0–336), MRVS = Modified Rush Video-Based Tic Scale (range: 0–20), PUTS = Premonitory Urge for Tics Scale (range: 0–36), GCI for PU = Global Clinical Impairment for premonitory urges (range: 0–100), YGTSS-GS = Global scale of the Yale Global Tic Severity Scale (range: 0–100), GCI = Global Clinical Impairment (range: 0–100), GTS-QOL = Gilles de la Tourette Syndrome-Quality of Life Scale (range: 0–100), GTS-QoL-VAS = Visual Analogue Scale for satisfaction of the GTS-QOL (range: 0–100).

Treatment with nabiximols resulted in an improvement of overall tic severity by 22.2% (YGTSS-TTS: pre = 45, post = 35) and 58.8% (MRVS: pre = 17, post = 7) according to examiner’s assessments, and by 62.9% according to the patient’s self-assessment (TSSL: pre = 89, post = 33). The improvement in YGTSS increased further when disease-related burden was included (35.2% YGTSS-GS: pre = 85, post = 55). Accordingly, GCI exhibited an improvement of 50% (pre = 80, post = 40). Using two different measurements for premonitory urges, we found substantial improvement as well (35.3% PUTS: pre = 34, post = 22, 38.5% GCI for PU: pre = 65, post = 40). However, the most predominant effects were observed for quality of life, which exhibited improvements of 78.4% and 70% according to GTS-QOL (pre = 51, post = 11) and GTS-QOL-VAS (pre = 50, post = 85), respectively. All results are summarized in Table 1 and illustrated in Figure 1. Treatment with nabiximols was well-tolerated by the patient and no noteworthy side effects were encountered. 

Soon after the follow-up visit, the patient stopped attending further consultations. Therefore, little can be said about further compliance and the long-term effects of nabiximols in this individual case.

## 4. Limitations

Tics usually follow a course of waxing and waning, therefore symptom improvement can never be reliably linked to a medication effect looking at a single case. However, in the case of our patient, who was incapacitated by his tics for a prolonged period of time, spontaneous substantial improvement was very unlikely to occur. Since this was an open trial, placebo effects cannot be distinguished from the medication effect. Still, we would not assume a large placebo effect in an otherwise treatment-resistant patient. Another limitation is that our patient was severely ill with a number of comorbid conditions and was thus phenotypically quite different from patients with a more common presentation of TS, which might limit the generalizability of our findings. In this study, we did not assess psychiatric comorbidities because at the time of treatment, tics were the core and most troublesome symptom of our patient and, therefore, treatment was initiated to reduce tics.

## 5. Discussion

Our case study is completely in line with another recent case report [14] and provides further evidence suggesting that treatment with nabiximols may be effective in the treatment of patients with severe and otherwise treatment-resistant TS. Therefore, our results corroborate recent findings in further open uncontrolled case studies [7,8,20,21,22,23] and small controlled clinical trials [9,10] reporting beneficial effects of CBM such as cannabis and THC in this group of patients. From currently available data, it is suggested that CBM may improve not only tics, but also psychiatric comorbidities including ADHD symptoms [7,20,23], obsessive-compulsive behavior [20,23], self-injurious behavior [7], impulse control [20], hypersexuality [7], as well as concentration and visual perception [21]. In this study, we concentrated on the treatment of tics and, therefore, did not assess behavioral problems. However, we found that the most predominant treatment effect was an improvement in the patient’s global impairment and quality of life. Therefore, it could be speculated that treatment with nabiximols in this patient resulted not only in an improvement of tics, but of comorbidities also. Comparable to other reports of treatment with CBM [7,20,23], our patient also experienced an improvement of premonitory urges during treatment with nabiximols. This is remarkable, since on the one hand the patient reports indicate that premonitory urges are perceived as the most troublesome symptom of the disease [24], but on the other hand an open uncontrolled study suggested that well-established treatment with antipsychotics such as aripiprazole improve tics, but not premonitory urges [25].

So far, there are no large controlled studies available investigating the effects of CBM in patients with TS or comparing the effects of CBM and antipsychotics or different CBM in TS. Given the positive response exhibited by the patient highlighted in this report and under the above-mentioned limitation, further investigation of the effect of nabiximols is strongly suggested. To properly investigate a treatment effect, a controlled clinical trial in a larger group of patients is called for. Therefore, our group is currently preparing a randomized multi-centre double-blind placebo controlled trial with 96 patients to investigate the efficacy and safety of nabiximols in the treatment of adults with chronic tic disorders (CANNA-TICS, EudraCT number: 2016-000564-42).

## 6. Conclusions 

Our case study suggests that treatment with nabiximols may be effective in patients with otherwise treatment-resistant severe TS. Large clinical trials are called for to further examine this hypothesis.

## Figures and Tables

**Table 1:** Clinical measurements before and after treatment with nabiximols.

Symptom	Scale	Baseline	Follow-Up after 2 Weeks	Percentage Improvement
Tics	YGTSS-TTS	45	35	−22.2%
	TSSL	89	33	−62.9%
	MRVS	17	7	−58.8%
Premonitory urges	PUTS	34	22	−35.3%
	GCI for PU	65	40	−38.5%
Global impairment	YGTSS-GS	85	55	−35.2%
	GCI	80	40	−50.0%
Quality of life	GTS-QOL	51	11	−78.4%
	GTS-QOL-VAS ^a^	50	85	70%

^a^ For GTS-QOL-VAS a positive percent change indicates improvement; for all other scales a negative change indicates improvement. Abbreviations: YGTSS-TTS = total tic score of the Yale Global Tic Severity Scale (range: 0–50), TSSL = Tourette Syndrome Symptom List (range: 0–336), MRVS = Modified Rush Video-Based Tic Scale (range: 0–20), PUTS = Premonitory Urge for Tics Scale (range: 0–36), GCI for PU = Global Clinical Impairment for premonitory urges (range: 0–100), YGTSS-GS = Global scale of the Yale Global Tic Severity Scale (range: 0–100), GCI = Global Clinical Impairment (range: 0–100), GTS-QOL = Gilles de la Tourette Syndrome-Quality of Life Scale (range: 0–100), GTS-QoL-VAS = Visual Analogue Scale for satisfaction of the GTS-QOL (range: 0–100).
